# Propranolol Reduces Portal Vein Diameter in Schistosomal Liver Disease with Portal Hypertension: A Prospective Cohort Study

**DOI:** 10.4269/ajtmh.19-0452

**Published:** 2020-02-17

**Authors:** Edford Sinkala, Michael Vinikoor, Kanekwa Zyambo, Ellen Besa, Bright Nsokolo, Paul Kelly

**Affiliations:** 1 Department of Internal Medicine, University Teaching Hospital, Lusaka, Zambia;; 2 Department of Internal Medicine, Tropical Gastroenterology and Nutritional Group, University of Zambia, Lusaka, Zambia;; 3 Department of Medicine, University of Alabama at Birmingham, Birmingham, Alabama;; 4 Centre for Infectious Disease Research in Zambia, Lusaka, Zambia;; 5 Blizard Institute, Barts and The London School of Medicine, Queen Mary University of London, London, United Kingdom

## Abstract

Hepatosplenic schistosomiasis (HSS) complicates portal hypertension, leading to life-threatening variceal bleeding. Variceal bleeding is associated with increased portal vein diameter (PVD). Beta-blockers prevent variceal bleeding. It is unclear whether beta-blockers such as propranolol can reduce PVD in HSS. We aimed to explore the effect of propranolol on PVD in HSS. A longitudinal study was conducted at the University Teaching Hospital, Zambia, as an extension of a clinical trial of rifaximin undertaken to test the hypothesis that rifaximin could reduce bacterial translocation in HSS. We randomized 85 adults to either rifaximin and standard care, or propranolol-based standard care only for 42 days. We then followed up all the patients on propranolol up to day 180. We used ultrasound to measure PVD at baseline and day 180. The primary outcome was reduction in PVD. Beta-blockade and splenic size reduction were secondary outcomes. Portal vein diameter reduced after 180 days of propranolol therapy from median 12 mm (interquartile range (IQR): 11–14) to median 10 mm (IQR: 9–13) (*P* < 0.001). The pulse rate reduced from baseline median 70 beats/minute (IQR: 66–80) to 65 beats/minute (IQR: 60–70) by day 180 (*P* = 0.006). Hemoglobin levels improved from baseline median 8 g/dL (IQR: 6–11) to 12 g/dL (10–14) (*P* < 0.001). Splenic size remained unchanged. Propranolol led to the reduction in PVD over 180 days. This suggests that ultrasound could be useful in monitoring response and compliance to beta-blockers, especially in resource-constraint areas where portal hypertension measurement facilities are unavailable.

## INTRODUCTION

Hepatosplenic schistosomiasis (HSS) is a leading cause of portal hypertension in the tropics, whereas cirrhosis is the dominant cause in Western countries.^1,2^ In Zambia, schistosomiasis is endemic in some areas, with seroprevalence as high as 88%.^3–5^ Exposure to water bodies through swimming, farming, and drawing water for domestic use accounts for acquisition of *Schistosoma mansoni* infection in an African setup.^6–9^ The most important cause of mortality in HSS is variceal bleeding, which is a direct consequence of portal hypertension.^6,10,11^ Variceal bleeding can be life-threatening,^2^ particularly in resource-limited settings with low capacity for diagnosis and interventional endoscopy coupled with challenges with blood supply for transfusion.

The pathophysiology of schistosomal portal hypertension is quite complex. The disease mainly causes periportal fibrosis as opposed to cirrhosis in which hepatocellular function is affected.^12–16^ Portal hypertension occurs when there is an increase in portal vein pressure and is diagnosed when the hepatic venous pressure gradient (HVPG) is greater than 5 mm Hg.^17^ In cirrhosis, an increase in HVPG of ≥ 10 mm Hg results in complications that include variceal bleeding, ascites, hepatorenal syndrome, and hepatic encephalopathy.^18–20^ In non-cirrhotic portal hypertension such as HSS, complications such as hepatorenal syndrome, coagulopathy, and hepatic encephalopathy are very rare.^21^ Measurements of portal pressure are invasive and quite sophisticated, and very few centers in Africa are able to offer this service because of inadequate expertise including lack of equipment. Assessment of portal vein diameter (PVD) using ultrasound may be used to predict portal hypertension. It was reported in 1982 that PVD greater than 13 mm was associated with portal hypertension.^22^ Beta-blockers are effectively used in primary and secondary prevention of variceal bleeding in cirrhosis and schistosomiasis-related portal hypertension.^23–25^ Propranolol, a beta-blocker, is able to reduce mortality in patients with schistosomiasis-related portal hypertension.^26^ Beta-blockers are able to prevent variceal bleeding by reducing splanchnic blood flow by causing vasoconstriction. In addition, they reduce cardiac output.^27,28^ Although beta-blockers are widely used, it is not clear whether they have an effect on PVD in schistosomiasis-related portal hypertension. We are not certain whether reduction in PVD is associated with reduction in episodes of variceal bleeding or not. We undertook a longitudinal prospective study to explore the effect of beta-blockade on PVD in HSS-related portal hypertension.

## MATERIALS AND METHODS

### Study design.

This was an observational prospective longitudinal study of HSS patients as an extension of a 42-day rifaximin clinical trial. The 42-day rifaximin clinical trial was undertaken to test the hypothesis that rifaximin could reduce markers of bacterial translocation in HSS.

### Study population.

Patients were evaluated and recruited at the University Teaching Hospital, Lusaka, Zambia. The recruitment took place in the Department of Internal Medicine and endoscopy unit between January 2014 and August 2016. The patients to be enrolled needed to have the following parameters as the inclusion criteria: 1) hematemesis and/or splenomegaly, 2) varices on endoscopy, 3) periportal fibrosis on ultrasound, 4) positive serology for schistosomiasis, and 5) ≥ 18 years of age. The exclusion criteria included ultrasound suggestive of cirrhosis (small shrunken liver with irregular margins); seropositivity for HIV, hepatitis B virus, and hepatitis C virus (HCV); or inability to consent.

### Study medications.

Of the 186 patients with portal hypertension who were evaluated, 85 were eligible and randomized to either rifaximin with standard care or standard care only. Forty-four (44) patients received rifaximin and standard care, whereas 41 received standard care only. After 42 days of the rifaximin clinical trial, both groups continued on standard care (with clinical visits every 2–4 weeks as routine) and were followed up until day 180. The standard care included oral propranolol, a beta-blocker, which was initiated at a dose of 40 mg three times daily and titrated upward, aiming at a resting radial pulse of ≤ 60 beats/minute. All the patients received praziquantel 40 mg per kg body weight orally in divided doses over a day.

### Evaluation.

All patients were to complete the 180-day follow-up. The primary outcome was the median change in PVD from enrollment to day 180. The secondary outcomes were beta-blockade (measured as a resting pulse of ≤ 60 beats/minute), change in splenic size, and mortality.

### Assessments.

A questionnaire was administered at baseline and on day 180 to capture demographic data, medical history, and social history. The patients also underwent a thorough physical examination at these two time points. Pulse was measured manually in the sitting position after the patient had rested for at least 5 minutes. Baseline and day 180 abdominal ultrasound measurements were performed to assess the size of PVD using a digital ultrasonic scanner (model P09, 2012, manufactured in Shenzen, China by Shenzen Landwind Industry Co. Ltd.). Abdominal ultrasound at the two time points also assessed the liver, splenic size, and presence of ascites. All the measurements were carried out by the same individual (E. S.) who was blinded to the baseline measurements.

Ten milliliter of blood was drawn and half of it was used for full blood count (Sysmex 800i analyzer, Koke, Japan) at baseline and after 180 days. The other half was centrifuged at 3,000 rpm, and plasma aliquoted in triplicates and stored at −80°C. Alanine aminotransferase and aspartate aminotransferase were analyzed using an HORIBA ABX Pentra 400 machine (Montipellier, France).

### Data analysis.

We used STATA version 13.1 (Stata Corp, College Station, TX) and GraphPad Prism 6.01 (GraphPad Software, San Diego, CA) for data analysis. To describe the data, we used median with interquartile range (IQR) as the data were not normally distributed. To compare measurements from baseline to day 180 within the group, we used the Wilcoxon matched-pairs sign-rank test. To compare baseline variables over the three groups (all patients, rifaximin group, and non-rifaximin group), the Kruskal–Wallis test was used. A *P*-value less than 0.05 was considered significant.

### Ethical consideration.

This study was approved by the University of Zambia Biomedical Research Ethics Committee (ref: 006-07-12). We sort informed consent verbally and in writing from all the participants.

## RESULTS

**Figure 1.**
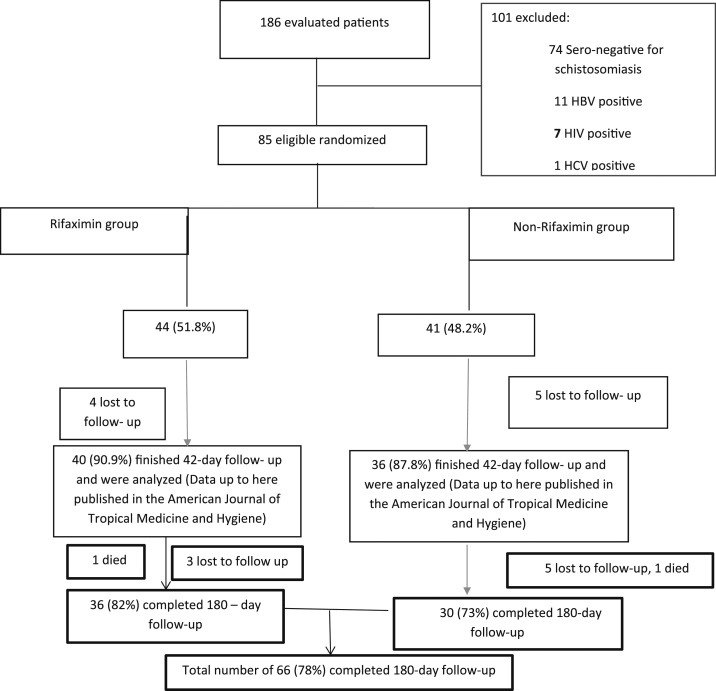
Study flowchart.

**Figure 2.**
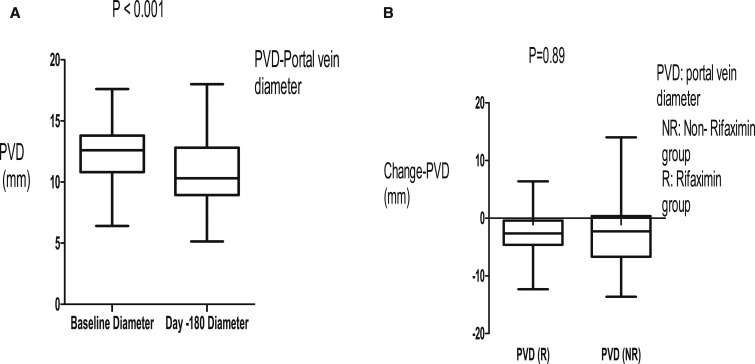
(**A**) Portal vein diameter (PVD) reduced after 180 days of follow-up on propranolol. (**B**) Change in PVD was similar in the rifaximin group and non-rifaximin group after 180 days of follow-up.

**Figure 3.**
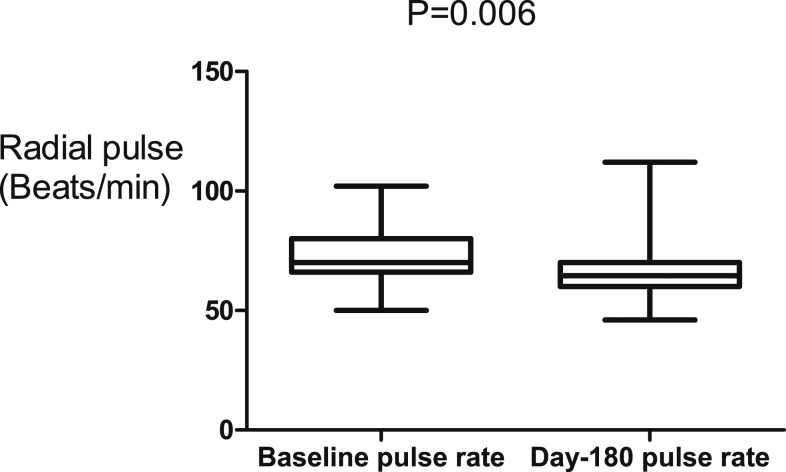
Propranolol reduced the radial pulse over the 180-day period.

**Figure 4.**
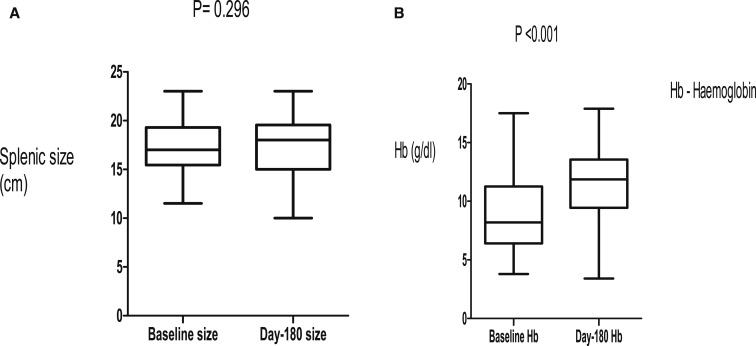
(**A**) Splenic size remained the same after 180 days of follow-up. (**B**) Hemoglobin levels increased after 180 days of follow-up.

In this study, 74 (40%) patients were excluded on the basis of negative serology for schistosomiasis, although all of them had periportal fibrosis on abdominal ultrasound scan (Figure 1). This was to maintain homogeneity in the study. Stool examination in our patients showed that only four patients were positive for *Schistosoma* ova. Of those who were enrolled, 19 (22%) did not complete the 180-day follow-up, and the outcome of these patients was not known (Figure 1). The three groups of patients with HSS were not different in relation to age, body mass index, pulse rate, and main PVD at baseline (Table 1). There was profound thrombocytopenia as part of pancytopenia across the groups (Table 1). These data showed that the patients across the three groups had massive splenomegaly, which was comparable (Table 1). There was no evidence of obesity in these patients (Table 1). Portal vein diameter and pulse rate reduced on propranolol therapy during the follow-up period (Figures 2 and 3). The PVD reduced from baseline, median of 12 mm (IQR: 11–14) to 10 mm (IQR: 9–13) by day 180 (*P* < 0.001). The change in PVD stratified by rifaximin and non-rifaximin groups over the period of 180 days was similar (Figure 2B). The pulse rate decreased from a baseline median of 70 beats/minute (IQR: 66–80) to 65 beats/minute (IQR: 60–70) (Figure 3) by day 180 (*P* = 0.006). Hemoglobin levels improved significantly from a baseline median of 8 g/dL (IQR: 6–11) to 12 g/dL (IQR: 10–14) (Figure 4B) by day 180 (*P* = 0.001). The splenic size was not affected during the follow-up (Figure 4A). Eight (9%) patients reported episodes of hematemesis during the follow-up. Nonadherence to propranolol during the follow-up was reported by 15 (18%) patients. Sixteen (19%) patients had ascites at baseline, whereas 12 (18%) of those who completed the 180-day follow-up had ascites.

**Table 1:** Baseline demographic, laboratory, and ultrasonographic data in hepatosplenic schistosomiasis patients

Variable	All patients, *n* = 85	Rifaximin group, *n* = 44	Non-rifaximin group, *n* = 41	*P*-value
Age (years)	40 (30, 48)	42 (30, 52)	38 (31, 43)	0.57
Body mass index (kg/m^2^)	22 (20, 23)	22 (20, 24)	22 (22, 23)	0.99
Pulse rate (beats/minute)	70 (66, 80)	76 (66, 82)	70 (64, 76)	0.37
Main portal vein diameter (mm)	12 (11, 14)	13 (11, 14)	12 (11, 15)	0.47
Splenic size (cm)	17 (15, 19)	17 (14, 19)	17 (16, 19)	0.57
Hemoglobin (g/dL)	8 (6, 11)	8 (6, 11)	9 (7, 11)	0.89
Platelet count (10^9^/L)	46 (29, 69)	49 (31, 78)	44 (24, 64)	0.53
White cell count (10^9^/L)	2 (1, 3)	3 (1, 4)	2 (1, 3)	0.31
Alanine aminotransferase (U/L)	28 (24, 42)	30 (27, 34)	26 (16, 61)	0.78
Aspartate aminotransferase (U/L)	45 (36, 63)	44 (40, 47)	46 (31, 82)	0.99

Two serious adverse events occurred during the follow-up. The first one was a female patient with diabetes mellitus on oral hypoglycemic drugs. She died on day 112 of the follow-up as a result of diabetic ketoacidosis complicating sepsis and renal failure. The second patient died from hypovolemic shock secondary to an acute massive variceal bleeding. He died on day 150 during the follow-up.

## DISCUSSION

Beta-blockers are an important integral part in managing schistosomiasis-related portal hypertension.^23,26,29^ Our data suggest that propranolol, a beta-blocker, is associated with reduction in PVD in patients with HSS. The main known mechanisms of propranolol in preventing variceal bleeding are its effects on reducing cardiac output and causing splanchnic vasoconstriction.^27,28,30^ The reduction in PVD could be an additional pharmacological mechanism of propranolol for reducing variceal bleeding. The observance of the reduction in PVD after 180 days of propranolol therapy may necessitate the use of abdominal ultrasound in monitoring response and compliance to beta-blockers and also predicting variceal bleeding in HSS, especially in resource-constraint places where invasive and sophisticated portal hypertension measurement facilities are not readily available. Ultrasound services are not universal in resource-constraint areas but are the most available and cheap imaging methods to diagnose HSS and other liver diseases in these areas. The PVD measurements using ultrasound were carried out by the same individual at baseline and at day 180 to ensure uniformity, and he was also blinded to the baseline measurements to reduce bias.

There was a significant reduction in the radial pulse rate after 180 days compared with baseline measurements. This is attributable to propranolol, which further reaffirms the effect of propranolol on PVD. Seventy-five percent (75%) of HSS patients were compliant to propranolol, and this is confirmed by the effective beta-blockade, which may explain the small number of variceal bleeding episodes during the follow-up.

In our cohort, some patients had ascites at baseline and this persisted up to day 180, although ascites is not a common feature of HSS.^21^ The presence of ascites may mean that our cases had advanced liver disease due to schistosomiasis. Propranolol is known to affect the hemodynamics of portal hypertension in cirrhosis by reducing portal pressure, but it has not been effective in the treatment of ascites.^31^ This may be applicable to schistosomiasis-related portal hypertension as well.

A negligible number of patients were positive for *Schistosoma* ova on stool examination, which may mean that many of our patients were infected remotely probably during childhood, and hepatic fibrosis was a long-term complication. The other long-term common complication of HSS is splenomegaly.^32,33^ The splenic size in our cohort remained unchanged after 180 days of follow-up. Although it occurs from time to time, reduction in splenomegaly is not a common occurrence in patients with portal hypertension due to HSS according to our experience. It was reported in 2015 that splenectomy is of clinical importance in patients with HSS as it leads to an increase in platelet count, improves hemostatic and liver function, and reduces portal pressure.^34^ In this study, none of the patients underwent splenectomy. The full blood count picture showed pancytopenia, which could be attributed to hypersplenism. Hemoglobin levels improved at the end of the follow-up, which may be attributed to the lower rates of variceal bleeding.

Even if this study was an extension of the 42-day rifaximin clinical trial, the adverse event (mortality) which occurred on day 112 of follow-up could not be attributed to rifaximin. Any rifaximin in the gut would have been excreted long before this adverse event occurred because it is a nonabsorbable antibiotic.^35–37^ The mortality that occurred because of massive variceal bleeding in the non-rifaximin group could be attributed to poor compliance to propranolol.

A good number of our patients were excluded on the basis of negative schistosomiasis serology even if they had periportal fibrosis on ultrasound. This was performed to maintain homogeneity in our study patients. Our focus was to study a well-characterized group of patients with schistosomiasis who fulfilled all the criteria. The limitations of this study were that the compliance to propranolol was self-reported and no propranolol blood levels were checked. Liver biopsies were not carried out. There are few conditions which may appear like periportal fibrosis on ultrasound. Tuberculosis rarely causes isolated periportal tuberculosis which may appear as periportal fibrosis.^38^ Febrile patients with enteric fever, brucellosis, acute viral hepatitis, and chronic HCV infection can present with mild hepatic periportal thickening.^39^ However, all our patients had normal axillary temperature and had no other constitutional symptoms.

In conclusion, the observance of the reduction in PVD in this cohort of HSS patients after 180 days of propranolol suggests that abdominal ultrasound may be useful in monitoring response and compliance to beta-blockers. This is necessary especially in resource-constraint areas where invasive and sophisticated portal hypertension measurement facilities are not readily available. It would also be useful to establish whether PVD can be used to predict episodes of variceal bleeding in patients with HSS.
